# Correlation of Liver Enzymes and Blood Parameters With Histopathological Severity in Benign and Malignant Liver Lesions

**DOI:** 10.7759/cureus.92954

**Published:** 2025-09-22

**Authors:** Umair Toqueer, Abdul Manan, Anila Riyaz, Aliya Zaman, Rubina Shafi, Roomisa Anis, Muhammad Kamran Khan, Razwan Ashraf, Shahzail Ahmad Qureshi

**Affiliations:** 1 Medicine, Pinderfields Hospital, Wakefield, GBR; 2 Otolaryngology, Worcestershire Acute Hospitals NHS Trust, Worcester, GBR; 3 Pathology, Ayub Medical College, Abbottabad, PAK; 4 Pathology, Muhammad Medical and Dental College, Mirpur Khas, Mirpur Khas, PAK; 5 Pathology, National University of Sciences and Technology (NUST) School of Health Sciences, Islamabad, PAK; 6 Biochemistry, Riphah International University, Islamabad, PAK; 7 Biochemistry, National University of Sciences and Technology (NUST) School of Health Sciences, Islamabad, PAK; 8 College of Medicine, Shaqra University, Shaqra, SAU; 9 Medicine, Aziz Bhatti Shaheed Teaching Hospital, Gujrat, PAK; 10 Medicine, American University of Barbados, Wildey, BRB

**Keywords:** hematological markers, hepatocellular carcinoma, histopathology, liver enzymes, liver lesions

## Abstract

Background: Liver lesions, whether benign or malignant, often present with nonspecific clinical features, making early diagnosis and prognostication challenging. Histopathological evaluation remains the gold standard, but serum biomarkers could offer non-invasive alternatives for assessing disease severity.

Objectives: This study aimed to evaluate the role of liver enzymes and hematological parameters in differentiating benign from malignant liver lesions and to correlate these markers with histopathological severity.

Methodology: A prospective observational study was conducted at Ayub Medical College, Abbottabad, Pakistan, from January to December 2024, involving 120 patients with radiologically confirmed liver lesions. Blood samples were analyzed for alanine aminotransferase (ALT), aspartate aminotransferase (AST), alkaline phosphatase (ALP), total and direct bilirubin, hemoglobin, leukocyte count, platelet count, and erythrocyte sedimentation rate (ESR) using standard laboratory protocols. Histopathological grading of malignant lesions was performed on hematoxylin and eosin (H&E)-stained sections. Independent t-tests and one-way analysis of variance (ANOVA) were applied to assess group differences, and Pearson’s correlation analysis was used to examine associations with tumor grade.

Results: Among 120 patients, 72 (60%) had malignant and 48 (40%) had benign liver lesions. Malignant lesions demonstrated significantly elevated ALT, AST, ALP, and bilirubin levels, along with lower hemoglobin and higher leukocyte counts and ESR (p < 0.001 for all). These parameters also showed significant progressive changes across low-, intermediate-, and high-grade malignancies. Strong positive correlations were found between histopathological grade and ALT (r = 0.61), AST (r = 0.68), ALP (r = 0.59), and ESR (r = 0.71), while hemoglobin showed a strong negative correlation (r = -0.65). Platelet count did not significantly differ between groups or correlate with severity.

Conclusion: Routine liver enzymes and hematological markers demonstrate significant diagnostic and prognostic value in evaluating liver lesions. Their integration into clinical workflows may enhance early detection, especially in resource-limited settings.

## Introduction

Liver lesions encompass a wide spectrum of benign and malignant conditions, which often present overlapping clinical and radiological features, making early and accurate differentiation a diagnostic challenge. Among malignant liver diseases, hepatocellular carcinoma (HCC) represents the most common primary hepatic malignancy, accounting for more than 70% of liver cancers globally, with risk factors such as chronic hepatitis B and C virus infections, cirrhosis, aflatoxin exposure, and genetic disorders playing key roles in its pathogenesis [1]. In contrast, benign liver lesions, such as hemangiomas, hepatic adenomas, and focal nodular hyperplasia, are frequently incidental findings and may not require intervention unless symptomatic or at risk of malignant transformation.

The asymptomatic nature of liver tumors, particularly in early stages, often delays diagnosis until the disease has progressed to an advanced, less treatable stage. As emphasized by Baraka et al., the only hope for effective intervention lies in early detection and accurate discrimination between malignant and benign lesions [2]. Conventionally, imaging and serum alpha-fetoprotein (AFP) levels have been employed for screening and diagnosis. However, AFP suffers from low sensitivity and specificity, as it can also be elevated in various non-malignant liver conditions such as viral hepatitis and cirrhosis [3]. Consequently, there is growing interest in exploring other serum biochemical markers and hematological indicators that may serve as adjuncts to histopathology for evaluating lesion severity and malignancy risk.

Liver enzymes such as alanine aminotransferase (ALT), aspartate aminotransferase (AST), and alkaline phosphatase (ALP) are routinely measured as indicators of hepatic injury or cholestasis. Their elevation has been correlated with inflammatory and neoplastic processes affecting the liver [4]. Similarly, hematological alterations, including anemia, leukocytosis, and elevated erythrocyte sedimentation rate (ESR), are commonly observed in malignancies and chronic liver diseases, reflecting systemic inflammation and hepatic dysfunction. Prior studies have suggested that these routinely available blood parameters may show significant variation across different types and grades of liver lesions, and could potentially reflect the underlying histological severity [5,6]. In this context, these markers may have a dual role: they can be explored as diagnostic indicators to differentiate benign from malignant lesions, as well as prognostic indicators to assess the severity and progression of malignant disease. Biologically, such parameters may capture both tumor burden (through enzymes released by hepatic tissue injury or cholestasis) and systemic inflammation (through hematological changes), thereby providing a broader reflection of disease activity.

Given the limitations of existing diagnostic tools, there is a compelling need to evaluate the diagnostic utility of conventional biochemical and hematological markers in the context of liver pathology. While several studies have examined individual markers such as tumor necrosis factor-alpha (TNF-α), epidermal growth factor (EGF), and glutathione-S-transferase alpha (GST-α) in the setting of liver cancer, few have systematically correlated routine liver function and blood parameters with histopathological grading of liver lesions [7].

Therefore, the rationale of this study is to bridge this gap by investigating whether standard liver enzymes and hematological indices can reliably reflect the histopathological severity of both benign and malignant liver lesions. The objective of this study is to assess the diagnostic and prognostic relevance of liver enzymes and blood parameters in correlation with histopathological findings in patients presenting with focal liver lesions.

## Materials and methods

This prospective observational study was conducted at the Department of Pathology, Ayub Medical College, Abbottabad, Pakistan, over a period of one year, from January 2024 to December 2024. The primary aim was to investigate the association between liver function enzymes and hematological parameters with the histopathological severity of benign and malignant liver lesions.

The study population included patients of all genders, aged 18 years and above, who presented with radiologically confirmed liver space-occupying lesions and underwent liver biopsy or surgical resection for histopathological diagnosis at Ayub Teaching Hospital, affiliated with Ayub Medical College. The inclusion criteria comprised patients with newly diagnosed focal liver lesions, either benign or malignant, confirmed by histopathological examination. Patients with a prior history of chronic liver disease, systemic malignancies, previous hepatic surgery, or those on hepatotoxic medications were excluded to minimize confounding factors.

The sample size was calculated using the formula for estimating proportions with a 95% confidence interval and 80% power, based on an anticipated prevalence of histopathological abnormalities in liver lesions (p = 0.5 to maximize the sample size). This yielded a minimum required sample size of 96 patients, and the study ultimately recruited 120 patients, thus exceeding the calculated requirement.

A non-probability purposive sampling technique was employed to recruit eligible participants from the surgical and radiology departments. All patients were thoroughly counseled, and informed written consent was obtained before inclusion in the study. The study protocol was reviewed and approved by the Ethical Review Board of Ayub Medical College, Abbottabad (Approval No: 1423/23; dated, 18-12-2023).

Each patient underwent a standardized diagnostic workup prior to histopathological evaluation. Blood samples were collected under aseptic conditions before the biopsy or surgery for biochemical and hematological investigations. Liver function tests were analyzed using the COBAS INTEGRA 400 plus analyzer (Roche Diagnostics, Mannheim, Germany), which included ALT, AST, ALP, total bilirubin, and direct bilirubin levels. These parameters were measured enzymatically based on photometric analysis. Hematological parameters such as hemoglobin concentration, total leukocyte count, platelet count, and ESR were assessed using an automated hematology analyzer (Sysmex XN-1000, Sysmex Corporation, Kobe, Japan), and ESR was measured using Westergren’s method.

All liver tissue specimens obtained either via ultrasound-guided core needle biopsy or surgical resection were fixed in 10% buffered formalin, processed routinely, and embedded in paraffin. Sections of 4-5 μm thickness were stained with hematoxylin and eosin (H&E) and reviewed independently by two experienced histopathologists. Lesions were categorized as benign (e.g., hemangioma, focal nodular hyperplasia, hepatic adenoma) or malignant (e.g., HCC, metastatic carcinoma, cholangiocarcinoma). Additionally, the severity of malignant lesions was graded based on cellular atypia, mitotic figures, and architectural distortion, following WHO classification guidelines.

Data were entered and analyzed using IBM SPSS Statistics for Windows, Version 26 (Released 2020; IBM Corp., Armonk, New York, United States). Continuous variables such as liver enzyme levels and hematological parameters were expressed as mean ± standard deviation. Categorical variables such as lesion type and histological grading were expressed as frequencies and percentages. The relationship between liver enzymes and blood parameters with histopathological severity was analyzed using independent t-tests for continuous variables and chi-square tests for categorical variables. Pearson’s correlation coefficient was employed to assess linear associations. A p-value of less than 0.05 was considered statistically significant.

## Results

A total of 120 patients with liver space-occupying lesions were enrolled during the study period. The mean age was 51.4 ± 13.2 years. Of these, 70 (58.3%) were male and 50 (41.7%) were female, yielding a male-to-female ratio of 1.4:1.

Histopathological analysis revealed 48 (40.0%) benign lesions and 72 (60.0%) malignant lesions. Within the malignant group, HCC accounted for 45 (37.5%) cases, followed by metastatic adenocarcinoma (20, 16.7%) and cholangiocarcinoma (7, 5.8%). Among benign lesions, hemangioma (20, 16.7%), focal nodular hyperplasia (16, 13.3%), and hepatic adenoma (12, 10.0%) were observed (Table 1).

**Table 1:** Demographic and Histopathological Profile of Study Participants (n = 120) Data are presented as n (%) or mean ± standard deviation (SD). Percentages were calculated based on the total sample size (n = 120). FNH: focal nodular hyperplasia; HCC: hepatocellular carcinoma

Variable	n (%) or Mean ± SD	
Total Patients	120 (100)	
Mean Age (Years)	51.4 ± 13.2	
Gender	Male	70 (58.3)
	Female	50 (41.7)
Lesion Type	Benign	48 (40.0)
	Malignant	72 (60.0)
Benign Subtypes	Hemangioma	20 (16.7)
	FNH	16 (13.3)
	Hepatic Adenoma	12 (10.0)
Malignant Subtypes	HCC	45 (37.5)
	Metastatic Adenocarcinoma	20 (16.7)
	Cholangiocarcinoma	7 (5.8)

Comparison of liver function test parameters between the benign and malignant groups revealed significantly elevated levels of ALT, AST, ALP, and bilirubin (both total and direct) in malignant lesions (p < 0.05). The results are summarized in Table 2.

**Table 2:** Comparison of Liver Enzyme Levels Between Benign and Malignant Liver Lesions Values are expressed as mean ± standard deviation (SD). An independent t-test was applied, and a p-value < 0.05 was considered statistically significant. ALT: alanine aminotransferase; AST: aspartate aminotransferase; ALP: alkaline phosphatase

Parameter	Benign (n = 48)	Malignant (n = 72)	t-value	p-value
ALT (U/L)	42.3 ± 15.1	78.6 ± 22.5	-9.7	<0.001
AST (U/L)	39.8 ± 14.7	85.2 ± 26.4	-11.1	<0.001
ALP (U/L)	168.4 ± 56.2	274.6 ± 78.3	-8.0	<0.001
Total Bilirubin (mg/dL)	0.9 ± 0.3	2.1 ± 0.8	-9.8	<0.001
Direct Bilirubin (mg/dL)	0.3 ± 0.2	1.0 ± 0.4	-11.4	<0.001

The hematological parameters also showed statistically significant differences between benign and malignant groups. Hemoglobin levels were significantly lower in patients with malignant lesions, while total leukocyte count and ESR were significantly higher. Platelet count did not show a statistically significant difference (p > 0.05). The data are presented in Table 3.

**Table 3:** Comparison of Hematological Parameters Between Benign and Malignant Liver Lesions Values are expressed as mean ± standard deviation (SD). An independent t-test was applied, and a p-value < 0.05 was considered statistically significant. ESR: erythrocyte sedimentation rate

Parameter	Benign (n = 48)	Malignant (n = 72)	t-value	p-value
Hemoglobin (g/dL)	13.4 ± 1.5	10.8 ± 1.9	8.0	<0.001
Total Leukocyte Count (/mm^3^)	7,100 ± 1,200	10,200 ± 2,400	-8.1	<0.001
Platelet Count (×10^9^/L)	218 ± 54	206 ± 61	1.3	0.186
ESR (mm/hr)	22 ± 10	48 ± 14	-11.0	<0.001

Among the malignant cases, histopathological severity was graded into low-grade (n = 18), intermediate-grade (n = 28), and high-grade (n = 26) malignancies. Grading was applied according to histopathological criteria specific to each tumor type (HCC, cholangiocarcinoma, and metastatic adenocarcinoma), ensuring comparability within the malignant cohort. A one-way analysis of variance (ANOVA) test revealed statistically significant differences in enzyme levels and hematological parameters across increasing grades of malignancy. The means and p-values are detailed in Table 4.

**Table 4:** Biochemical and Hematological Parameters Across Histopathological Grades of Malignancy Values are expressed as mean ± standard deviation (SD). One-way analysis of variance (ANOVA) was applied, and a p-value < 0.05 was considered statistically significant. ALT: alanine aminotransferase; AST: aspartate aminotransferase; ALP: alkaline phosphatase; ESR: erythrocyte sedimentation rate

Parameter	Low Grade (n = 18)	Intermediate (n = 28)	High Grade (n = 26)	F-value	p-value
ALT (U/L)	62.8 ± 12.4	77.6 ± 15.8	91.4 ± 19.3	17.5	<0.001
AST (U/L)	68.2 ± 15.2	86.1 ± 20.7	103.2 ± 22.9	19.8	<0.001
ALP (U/L)	232.4 ± 41.6	273.2 ± 53.3	311.5 ± 62.8	14.2	<0.001
Total Bilirubin (mg/dL)	1.5 ± 0.5	2.2 ± 0.6	2.7 ± 0.7	16.1	<0.001
Hemoglobin (g/dL)	11.8 ± 1.2	10.5 ± 1.3	9.6 ± 1.5	14.6	<0.001
Total Leukocyte Count (/mm^3^)	8,900 ± 1,400	10,300 ± 1,700	11,600 ± 2,100	12.5	<0.001
ESR (mm/hr)	34 ± 8	48 ± 10	59 ± 12	20.3	<0.001

Pearson’s correlation analysis demonstrated a strong positive correlation between histopathological severity score and ALT (r = 0.61, p < 0.001), AST (r = 0.68, p < 0.001), ALP (r = 0.59, p < 0.001), and ESR (r = 0.71, p < 0.001). Hemoglobin levels were inversely correlated with malignancy grade (r = -0.65, p < 0.001). Platelet count showed no significant correlation (r = -0.12, p = 0.24).

These findings indicate that liver enzymes and certain blood parameters (especially ALT, AST, ALP, ESR, and hemoglobin) may serve as significant indicators of histopathological severity in patients with malignant liver lesions (Table 5).

**Table 5:** Pearson’s Correlation of Biochemical and Hematological Parameters With Histopathological Severity in Malignant Liver Lesions (n = 72) Pearson’s correlation test was applied. Data are presented as the correlation coefficient (r). A p-value < 0.05 was considered statistically significant. ALT: alanine aminotransferase; AST: aspartate aminotransferase; ALP: alkaline phosphatase; ESR: erythrocyte sedimentation rate

Parameter	r	p-value	Interpretation
ALT (U/L)	0.61	<0.001	Strong positive correlation
AST (U/L)	0.68	<0.001	Strong positive correlation
ALP (U/L)	0.59	<0.001	Moderate-to-strong positive
Total Bilirubin (mg/dL)	0.53	<0.001	Moderate positive correlation
Direct Bilirubin (mg/dL)	0.49	<0.001	Moderate positive correlation
Hemoglobin (g/dL)	-0.65	<0.001	Strong negative correlation
Total Leukocyte Count (/mm^3^)	0.55	<0.001	Moderate positive correlation
Platelet Count (×10^9^/L)	-0.12	0.240	No significant correlation
ESR (mm/hr)	0.71	<0.001	Strong positive correlation

## Discussion

This prospective study aimed to evaluate the diagnostic significance of liver enzymes and hematological parameters in distinguishing benign from malignant liver lesions, as well as to explore their correlation with histopathological severity. The results demonstrated that ALT, AST, ALP, total and direct bilirubin, hemoglobin, total leukocyte count, and ESR varied significantly between benign and malignant lesions and also across histological grades of malignancy, supporting their potential role as accessible biochemical indicators in hepatic lesion stratification.

In the present study, ALT and AST levels were significantly elevated in malignant liver lesions compared to benign ones (p < 0.001), with a progressive rise corresponding to increasing tumor grade. This is consistent with the findings of Noh et al. [8], who reported elevated transaminases in HCC patients, attributing this to hepatocellular necrosis and inflammatory cytokine release, especially TNF-α. The strong positive correlation between transaminases and histological severity in our study (ALT: r = 0.61; AST: r = 0.68) further supports this hypothesis, suggesting that enzyme elevation may not only indicate hepatocellular injury but also reflect the biological aggressiveness of malignant transformation. Zhang et al. [9] had previously linked elevated AST levels to the activation of Kupffer cells and increased TNF-α and IL-1 production, promoting hepatic inflammation and cellular damage.

Similarly, ALP was significantly higher in malignant lesions than in benign ones, and its levels increased in line with histopathological severity (p < 0.001). Elevated ALP in hepatic malignancy is typically associated with biliary obstruction, cholestasis, and tumor infiltration of bile ducts. Noh et al. [8] also reported significantly higher ALP levels in patients with HCC compared to those with other chronic liver diseases. The moderate-to-strong correlation between ALP and severity (r = 0.59) in our findings suggests its utility in reflecting tumor progression, particularly in biliary-invasive phenotypes like cholangiocarcinoma.

Bilirubin levels (total and direct) also showed marked elevation in malignant lesions, increasing with higher grades of malignancy. This observation can be attributed to impaired hepatic excretion, biliary compression, or direct tumor invasion. Similar findings were noted by Noh et al., who observed higher bilirubin levels in cirrhosis and HCC groups compared to controls and other benign liver diseases [8]. Although bilirubin is a less specific marker due to its elevation in various hepatic disorders, its progressive rise across malignancy grades supports its relevance in indicating hepatic functional deterioration in tumor-bearing states.

Hematological markers also provided important discriminatory insights. Hemoglobin levels were significantly lower in malignant cases (p < 0.001), with a strong inverse correlation to histological grade (r = -0.65). This aligns with the concept of anemia of chronic disease, common in malignancies due to chronic inflammation, bone marrow suppression, and nutritional deficiencies. Similar findings were reported by Spampinato et al. [10], who demonstrated hematological derangements in patients with liver cirrhosis and HCC, likely secondary to cytokine-mediated effects on erythropoiesis.

Leukocyte counts were significantly higher in malignant lesions and increased progressively with severity (r = 0.55). This leukocytosis may reflect tumor-associated inflammation, necrosis, or secondary infection. Elevated white cell counts are often observed in aggressive hepatic malignancies and correlate with inflammatory cytokine levels, particularly TNF-α and IL-6 [11]. Our results are consistent with these previous observations, reinforcing the role of systemic inflammation in malignancy progression.

The ESR emerged as one of the most strongly correlated parameters with histopathological severity (r = 0.71, p < 0.001). ESR is a nonspecific but sensitive indicator of systemic inflammation, and its elevation in malignancy is well-documented. The study by Jeng et al. [12] highlighted the relationship between inflammatory cytokines like TNF-α and disease severity in HCC, supporting the idea that elevated ESR reflects cytokine-driven systemic responses associated with tumor burden and necrosis.

Interestingly, platelet count did not differ significantly between benign and malignant lesions (p = 0.186) and showed no significant correlation with histological severity (r = -0.12). While thrombocytopenia is often reported in cirrhosis due to splenic sequestration and reduced thrombopoietin production, its behavior in primary liver malignancy without cirrhosis is less predictable. Our findings suggest that platelet count alone may not be a reliable discriminator of lesion type or severity, a conclusion supported by Noh et al. [8], who found weak diagnostic performance for platelet-related markers in liver disease.

Comparing our results with the study by Noh et al. [8], several parallels are evident. They emphasized the role of TNF-α, GST-α, and AFP in differentiating HCC from benign liver conditions, reflecting the importance of inflammation and oxidative stress in hepatocarcinogenesis. While our study focused on routine clinical parameters rather than specialized markers, the patterns of enzyme elevation and inflammatory response are in line with their conclusions. Both studies support the concept that serum biochemical profiles reflect underlying hepatic pathology and can serve as surrogate markers of histopathological change.

In contrast, our study provides the novel insight of correlating these biochemical markers directly with histopathological grading, revealing progressive alterations in ALT, AST, ALP, bilirubin, hemoglobin, leukocyte count, and ESR with increasing severity. This adds a prognostic dimension to their diagnostic utility, suggesting their potential role in non-invasive staging of hepatic malignancies.

This study was conducted at a single tertiary care center with a relatively limited sample size, which may restrict the generalizability of the findings. Additionally, the study focused on conventional biochemical and hematological parameters, without integrating advanced molecular or imaging biomarkers that may offer higher specificity. Histopathological grading was limited to H&E-based classification without the use of immunohistochemistry or genomic profiling for deeper insight into tumor biology.

The results demonstrate that routinely available liver enzymes and hematological parameters can provide valuable, non-invasive indicators of both the presence and severity of liver malignancies. These markers can aid clinicians in prioritizing patients for early histopathological evaluation, improve preoperative risk stratification, and support clinical decision-making in resource-limited settings where access to advanced diagnostics may be limited.

This study has certain limitations. First, it was conducted at a single tertiary care center with a relatively modest sample size, which may limit the generalizability of the findings. Second, the analysis relied solely on conventional biochemical and hematological parameters without incorporating advanced molecular or imaging biomarkers that could provide higher diagnostic specificity. Third, histopathological grading was performed using routine H&E staining without immunohistochemistry or genomic profiling, which might have offered deeper insight into tumor biology. Lastly, the cross-sectional design precluded longitudinal assessment of biomarker changes during disease progression or treatment response.

Future research should aim to validate these findings in larger, multicenter cohorts and explore the integration of conventional parameters with novel biomarkers such as AFP variants, GST-α, TNF-α, and genomic markers. Longitudinal studies assessing changes in these parameters during treatment and follow-up may also help in monitoring disease progression and response to therapy.

## Conclusions

This study highlights the significant association of liver enzymes and hematological parameters with the histopathological severity of benign and malignant liver lesions. Elevated levels of ALT, AST, ALP, bilirubin, leukocyte count, and ESR, along with reduced hemoglobin, were significantly correlated with increasing tumor grade. However, these findings should be interpreted with caution, as associations do not imply causation, and potential confounders, such as background liver disease or systemic inflammation, may have contributed to the observed patterns. These results underscore the potential utility of these accessible markers in the diagnostic and prognostic evaluation of liver lesions. Nonetheless, validation in larger, multicenter, and longitudinal studies is required before these parameters can be confidently integrated into standard clinical workflows.
